# A Few Practical Points in Certification

**Published:** 1909-05-22

**Authors:** 


### Mental Diseases.

### A FEW PRACTICAL POINTS IN CERTIFICATION.

The care of the insane being a matter of considerable expense and generally of long duration, if ny member of a family of moderate or small neans becomes insane, that individual frequently falls under the definition of a Pauper which is given in the Lunacy Act.

Now, every person whose work or inclination brings him into contact with all sorts and conditions of mankind, is bound to realise the hardsh ) of dressing in pauper costume and of placing in the same wards with the lowest and pauper classes those who are, and who always have been, respectable in life, and who are of fair or good education. These latter, no matter what their occupation, do not habitually mix with the lower class, and it is only misfortune which can force them to do so. Owing, however, to lack of space and of other conditions necessary for proper classification in public asylums, all classes of patients in them have to be more or less mixed together, since each patient has one circumstance in common with the rest—namely, lack of means to pay more than the cost of maintenance. The mental pain experienced by recovering patients of the better class, when they become conscious of their surroundings and find themselves in pauper clothes, is often seen n county asylums; and this mental shock very requently interrupts recovery, if it does not lead o further breakdown.

With insane patients of this class therefore, it is always well to advise application for admission to registered hospital before allowing them to be nt to the county asylum. Should the case beme suddenly urgent and removal from home ecessary before the hospital has agreed to take it, / proper application to a constable, relieving fficer, or overseer, the patient can be removed for he time being to the workhouse infirmary, under Section 20 of the Lunacy Act When the patient s removed to a registered hospital he is certified as a private patient, and the usual certificates—a petition, two medical certificates, and a justice's order—are needed. If a vacancy cannot be secured at a registered hospital, the county asylum is the only alternative. Private patients are admitted at most county asylums on payment of from 15s. to 25s. a week, the patient being kept, if possible, in the better wards and wearing their own clothes.

Sometimes the authorities of a county asylum vill refuse the application to admit as a private patient on payment of the required fees. When his happens the matter can often be settled by btaining the admission of the patient as a pauper nd by renewing, soon after admission, the offer of he required fees. The authorities then find themelves in a dilemma, and have either to do what hey before refused, and to transfer the patient to he "private" class, or to refuse a weekly sum of noney above the cost of maintenance. The patient vill usually be transferred forthwith to the private lass.

When the necessary arrangements have been made for admission, and it is essential that the patient be removed at once, this may be done on an urgency order. The urgency order must be signed, if possible, by the husband, wife, or a near relative of the patient; and if this is not possible, the reason must be stated. The person signing the urgency order must have seen the patient within two days of so signing. A statement of particulars and a medical certificate accompany the order. The practitioner signing the medical certificate must not be related to, nor be partner or assistant to, the person signing the order; he must have personally examined the patient within two days of signing, and must state his reasons for considering the case to be urgent. The urgency order remains in force for seven days, by the expiration of which time the full certificates have to be obtained.

The full certificates for a "private" patient consist of a petition for an order, two medical certificates, and the judicial reception order. The petition must be signed, if possible, by the husband, wife, or a relative of the patient, who must have personally seen the patient within fourteen days of the presentation of the petition. This petition is accompanied by a statement of particulars. The two practitioners signing the medical certificates must have examined the patient, separately from each other and from any other practitioner, within seven clear days of the presentation of the petition, and must not be related to one another nor to the petitioner, nor must either be partner or assistant to the other nor to the petitioner. No medical certificate may be founded entirely on facts communicated by others, but must contain facts observed at the time of examination. It is not essential, however, to put in any facts communicated by others. When the petition and medical certificates are duly filled in and signed, they are taken with the order to a Justice specially appointed under the Act, to a judge of County Courts, or to a stipendiary magistrate, for his signature. The Justice signing the order need not necessarily see the patient, but has to state whether or not he has so seen him. The date of the petition should be the same as the date of the order. The reception order ceases to be valid unless the patient is removed to the institution mentioned therein within seven clear days from the date it was signed.

In the case of a patient who has to be certified as a pauper and removed to the county asylum, the method of procedure is to notify the relieving officer or overseer of the parish that the patient is insane. The person so notified has then to act within three days. If the case is urgent and the patient is not under proper care and control, the facts should be stated to the relieving officer that he may deal with it at once, by removing it to the infirmary. In this case the medical man should also state in writing that the patient is in a fit state of bodily health to be removed.

